# Extended Target Echo Detection Based on KLD and Wigner Matrices

**DOI:** 10.3390/s19245385

**Published:** 2019-12-06

**Authors:** Dingsu Xie, Fei Wang, Jun Chen

**Affiliations:** 1 Key Laboratory of Radar Imaging and Microwave Photonics, Nanjing University of Aeronautics and Astronautics, Ministry of Education, Nanjing 210016, China; dingsuxie@163.com; 2 School of Electronic and Information Engineering, Nanjing University of Information Science and Technology, Nanjing 210044, China; junchen@nuist.edu.cn

**Keywords:** radio frequency stealth (RFS), wideband radar, extended target, echo detection, Kullback–Leibler Divergence (KLD), Wigner matrix

## Abstract

With the development of airborne radar radio frequency stealth (RFS) technology, the method of improving the RFS performance of airborne radar by optimizing target detection performance has been extensively studied. However, for wideband radar signals, the traditional point target model appears as an extended target model in the range-dimension, which is unfavorable to the detection of target echoes. To overcome the existing drawbacks, this paper devises an efficient echo detection algorithm from the perspective of information theory and random matrix. Firstly, aperiodic agile wideband radar signals are utilized to observe targets. Then, one frame of echo signals in the same range gate is reconstructed into a data form conforming to the Wigner matrix spectral decomposition. Finally, according to the signal detection theory, Kullback-Leibler Divergence (KLD) is used as the test statistic to complete the echo detection of the stealthy extended targets. By statistical analysis and comparison with other established echo detection algorithms, simulation results manifest that the proposed algorithm has superior detection performance and strong robustness, which not only makes up for the deficiency of traditional narrowband radar detection algorithms, but also increases the detection probability of radar system when it is faced with stealthy extended targets.

## 1. Introduction

Radar RFS is overwhelmingly important in electronic war (EW), and waveform design and selection [1]. Low radiation power control is one of the main ways to realize RFS, which is more effective by improving the performance of detection algorithms to reduce the signal-to-noise ratio (SNR) of target detection. Radar target detection refers to the whole process of extracting target information by the radar receiver from the received target echo via suppressing the noise clutter.

Traditionally, radar RFS target detection includes digital beam forming (DBF), intrapulse matching filtering, pulse integration, and constant false alarm rate (CFAR) detection. Pulse integration can be divided into coherent integration and noncoherent integration, which have been widely studied by myriad scholars. The principal difference between the two approaches lies in that coherent integration takes into account the phase information of the target, so that the accumulation effect is better than the noncoherent integration. A typical coherent integration algorithm is the moving target detection (MTD) algorithm [2], which is implemented by fast Fourier transform (FFT) in a single range unit. Although the most effective way to achieve target detection at low SNR is to extend the target accumulation time, long-term observations can cause the target echo envelope to present across range unit (ARU) and Doppler frequency migration (DFM) phenomena [3,4], which seriously restrict the long-term accumulation performance of the target.

Aiming at the problem of ARU, since Zhang et al. applied Keystone transform (KT) for synthetic aperture radar (SAR) to target detection of pulse Doppler (PD) radar, KT has received extensive attention in radar target detection [5]. The second-order KT [6] can address the second-order ARU phenomenon prompted by the target acceleration. Subsequently, Kong et al. presented a generalized KT to cope with the ARU phenomenon of any order [7], and later scholars continue to bring forward KT for joint processing of Doppler fuzzy correction [8]. Aiming at the issue of DFM, time-frequency methods are considered first, including Wigner-Ville distribution and Choi-Williams distribution [9]. However, due to the existence of cross-terms, the two methods are not conducive to the parameter estimation of the signal. In that case, methods for suppressing cross-terms such as Radon-Wigner transform, Radon-ambiguity transform, and Wigner-Hough transform [10,11] are advocated. In recent years, target detection algorithms based on normality tests have also been put forward, including Jarque-Bera test, Lilliefors test, and Anderson-Darling test [12].

One attractive problem in radar systems is waveform design for the alleged “extended targets”, which are also referred to as “range-spread targets” in some studies [13]. Owing to a suitable reformulation of the considered nonconvex design problem, Cheng et al. devised an iterative optimization procedure over the transmit signal and the receive filter bank [14]. Ciuonzo et al. dealt with the problem of adaptive multidimensional/multichannel signal detection in homogeneous Gaussian disturbance with unknown covariance matrix and structured (unknown) deterministic interference [15]. Conte et al. addressed the problem of adaptive detection of range-spread targets in homogeneous and partially homogeneous environment [16]. It is well known that the range resolution is inversely proportional to the signal bandwidth. Compared with the narrowband radar, the wideband radar can offer the information of the distance, size, and shape of a target and form a high range resolution profile (HRRP). Dai et al. proposed and analyzed an adaptive detection method of range spread targets with range walking across range cells [17]. D. Ciuonzo also devised imaging functions for wideband computational time-reversal (C-TR) based on generalized likelihood ratio (GLR), Rao, and Wald statistics under the single-source model [18].

While the above-mentioned target detection algorithms provide us with a guidance to address the issue of low radiation power control, they still cannot handle the problem of target detection in low SNR. In this paper, as far as the limited sampling number of echo signals be concerned, we make some prospective research on the echo detection of stealth extended target of wideband radar signals from the perspective of information theory and random matrix.

In view of the aforementioned problems, in this paper, we present echo detection algorithm of stealth extended targets based on KLD and Wigner matrices. The spectral distribution of Wigner matrices in finite dimensions was taken as the essential feature of background white noise, and the signal detection was conducted by testing the similarity between the empirical spectral cumulative distribution function (CDF) of received signals and the spectral CDF of Wigner matrices in finite dimensions. The algorithm in this paper uses KLD as the test statistic of signal, which has a superior detection performance and robustness.

The major contributions of the proposed work are summarized as follows:

- Most of the current research assumes that the target is a “point target” model, but this is only applicable to the situation that the radar range resolution unit is much larger than the geometrical dimension of the target and the target scattering energy is concentrated in one range unit. In practical applications, especially for wideband radar signals, the range resolutions are relatively high, and the target scattering centers are distributed in multiple range units. If the traditional method is still adopted, especially the stealth target’s echo is exceedingly weak. This paper comes up with an echo detection algorithm for this sort of stealthy extended target.
- In this paper, the white Gaussian noise signal sequence in the environment is reconstructed into Wigner matrices, and the spectral distribution of Wigner matrices in finite dimensions is innovatively brought forward as the characteristic of white Gaussian noise. The KLD of the empirical spectral CDF and the finite dimensional spectral CDF of the reconstructed echo signal is calculated and used as the test statistic.
- Numerous studies on target detection are based on the assumption that the echo signal can be sampled sufficiently. However, the sampling frequency of actual radar receiver is limited. In this paper, the probability density function (PDF) and CDF of Wigner matrices are studied for the limited number of samples, and the properties of spectral distribution of Wigner matrices in finite dimensions are rigorously derived.
- Numerical results are provided to demonstrate that the proposed algorithm effectively improves the signal detection performance and is suitable for different low probability of intercept (LPI) radar waveforms. The method advocated in this paper can reduce the SNR required for radar target detection and achieve low radiation power control, so as to improve the RFS performance of airborne radar.

The rest of this paper is organized as follows. Section 2 introduces the application of KLD and Wigner matrices in echo detection. They are the core theoretical bases of the detection algorithm presented in this paper. In Section 3.1, the schematic of the proposed algorithm is expounded. Assuming that the echo signal has no target, we set forth the calculation of spectral distribution of Wigner matrices in finite dimensions constructed via the white Gaussian noise signal in Section 3.2. We make a rigorous derivation for the test statistic and detection threshold of the proposed algorithm in Section 3.3, whose performance is assessed at length via simulation presented in Section 4.1 and whose superiority in terms of detection performance compared to other classical methods is illustrated by comparative numerical results. Section 4.2 makes a detailed analysis of the influencing factors of detection performance. We present our concluding remarks in Section 5.

*Notation*: Vectors (matrices) are denoted by boldface lower (upper) case letters. The symbols ⊗, ⇒, ∑(·), Γ(·), and R represent Kronecker product, mapping, accumulation, Gamma function, and the space of real numbers, respectively. The cardinality of set A is denoted by #A. $E_{q}$ means that the random variable is averaged with *q*. Superscript ${\left(\cdot \right)}^{T}$ denotes transpose and $x_{n}\to l$ means that simple convergence of the series $x_{1},x_{2},\cdots$ to *l*.

## 2. Application of KLD and Wigner Matrices in Echo Detection

### 2.1. Application of KLD in Echo Detection

Since Shannon [19] published his paper “A mathematical theory of communication” in 1948, an army of scholars have also applied information theory to radar systems. One of the most representative results is that of Bell [20], who applied mutual information to radar waveform design in 1993. Numerous scholars make the broad range of research on the target detection, recognition, and tracking of radar waveforms based on the mutual information and KLD in the information theory. Extensive research by serious scholars establish the relationship between “entropy” and “radar signal processing”.

In radar systems and passive detection systems, the KLD between the received signal and the background noise of the radar/passive detection device is adopted to measure the receiving performance of the receiving system of the radar/passive detection device. KLD, which is also called “relative entropy”, is a measure of the difference between two probability distributions [21]. For instance, the KLD between PDF $q_{1}\left(x\right)$ and $q_{2}\left(x\right)$ can be defined as:

(1) $$D_{KL}\left(\left.q_{1}\right|\left|q_{2}\right.\right)=\int_{-\infty }^{\infty }q_{1}\left(x\right)\log\left[\frac{q_{1}\left(x\right)}{q_{2}\left(x\right)}\right]dx=E_{q_{1}}\left[\log\frac{q_{1}\left(x\right)}{q_{2}\left(x\right)}\right].$$

$D_{KL}\left(\left.q_{1}\right|\left|q_{2}\right.\right)$ is a non-negative binary function. Only when $q_{1}\left(x\right)=q_{2}\left(x\right),$ $D_{KL}\left(\left.q_{1}\right|\left|q_{2}\right.\right)=0$ can be established. Small $D_{KL}\left(\left.q_{1}\right|\left|q_{2}\right.\right)$ indicates $q_{1}\left(x\right)$ is similar to $q_{2}\left(x\right)$ [22]. Compared with the established methods used to measure the difference between PDFs, namely Bhattacharyya distance, F-divergence, and Hellinger distance, KLD has advantages of low computational complexity, clear physical concept, and rigorous mathematical derivation.

Assuming that the radar waveform is represented by $s\left(t\right)$ and the sampling frequency of the receiver is formulated as $f_{s}$, we set forth that the time-domain sampling value of the signal can be described as:

(2) $$s\left(n\right)=s\left(\frac{n}{f_{s}}\right),\;\;n=1,\ldots ,N,$$

where *N* denotes a finite number of samples.

The signal processed by the intercepted receiver is a signal in additive noise, which can be expressed by:

(3) $$r_{l}\left(n\right)=s\left(n\right)+w_{l}\left(n\right),\;\;n=1,\ldots ,N,\;\;l=1,\ldots ,L,$$

where $w_{l}\left(n\right)$ represents the sampled value of the additive white Gaussian noise (AWGN) with a mean of μ and a variance of $\sigma^{2}$. *L* represents the number of signals in additive noise.

**Figure 1:**
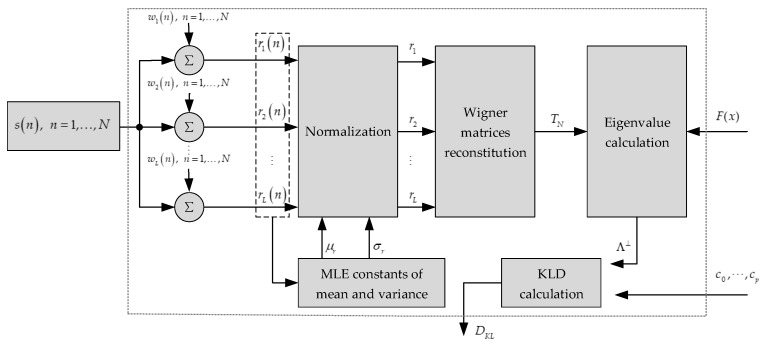
Calculation block diagram of radar RFS evaluation index KLD.

The KLD between the empirical spectral CDF $\left(F^{†}\left(x\right)\right)$ and the finite dimensional spectral CDF $\left(F\left(x\right)\right)$ is used as an airborne radar RFS performance indicator [23,24]. Its calculation process is depicted in Figure 1.

The KLD of $F^{†}\left(x\right)$ from $F\left(x\right)$ is defined as:

(4) $$D_{KL}\left(F\|F^{†}\right)=\int_{-\infty }^{+\infty }f\left(x\right)\log\left(\frac{f\left(x\right)}{f^{†}\left(x\right)}\right)dx,$$

where *f* and $f^{†}$ denote the PDF of *F* and $F^{†}$, respectively.

Since the interval of eigenvalues of random matrix $T_{N}$ is inconclusive, to simplify Equation (4), the probability integral transform $\lambda_{n}^{⊥}=F\left(\lambda_{n}\right)$ is used to transform the original eigenvalues to an equivalent set $\Lambda^{⊥}=\left\{\lambda_{n}^{⊥},n=1,\ldots ,N\right\}$. As $r_{l}\left(n\right)=w_{l}\left(n\right)$ and L→∞, the elements of set $\Lambda^{⊥}$ come from the uniform distribution U[0,1]. Thus, as set $\Lambda \Rightarrow \Lambda^{⊥}$, we have the density function $f\left(x\right)\Rightarrow u\left(x\right)\;,$ where $u\left(x\right)$ is given by:

(5) $$u\left(x\right)=\left\{\begin{array}{c} 1,\;x\in \left[0,1\right].\\ 0,\;else, \end{array}\right.$$

We also have the empirical spectral CDF $F^{†}\left(x\right)\Rightarrow U^{†}\left(x\right)=\frac{1}{N}\#\left\{n\leq N,\lambda_{n}^{⊥}\leq x\right\}$.

As previously stated, the KLD $D_{KL}\left(F\|F^{†}\right)$ can be calculated by:

(6) $$D_{KL}\left(U\|U^{†}\right)=\int_{-\infty }^{+\infty }u\left(x\right)\log\left(\frac{u\left(x\right)}{u^{†}\left(x\right)}\right)dx=-\int_{0}^{1}\log\left(u^{†}\left(x\right)\right)dx,$$

where $U^{†}$ is the empirical CDF of eigenvalue set $\Lambda^{⊥}$, *U* is the CDF of the uniform distribution in $\left[0,1\right]$, and $u^{†}\left(x\right)$ and $u\left(x\right)$ are the corresponding PDFs.

We define $c_{p},p=0,1,\ldots ,P$ as the partition points in the interval $\left[0,1\right]$, and $0=c_{0}<c_{1}<\cdots <c_{P}=1$. When P→∞ and $\max_{1\leq p\leq P}\to 0$, the test statistic $D_{KL}\left(U\|U^{†}\right)$ can finally be derived as:

(7) $$D_{KL}\left(U\|U^{†}\right)\approx -\sum_{p=1}^{P}\left(c_{p}-c_{p-1}\right)\log\left(\frac{U^{†}\left(c_{p}\right)-U^{†}\left(c_{p-1}\right)}{c_{p}-c_{p-1}}\right).$$

### 2.2. Application of Wigner Matrices in Echo Detection

With the increase of the number of antennas received by radar or passive sensor and the sampling data of each antenna, the data we need to process usually have the traits of high dimension. However, the traditional limit theorem is only applicable to data with fixed dimensions, and it is no longer applicable to data with higher dimensions [25]. Therefore, random matrix theory (RMT), which is used to process large-dimensional data, has been widely concerned and applied. Since E. Wigner established the well-known semicircular law, RMT has been developed into a pivotal research area in mathematical physics and probability. This paper makes proper use of the semicircular law property of the RMT to derive the essential characteristics of the signal.

An n×n Hermitian matrix $W_{n}$ is a Wigner matrix if its upper-triangular entries are independent zero mean random variables with identical variance. If the variance is 11nn, $W_{n}$ is a standard Wigner matrix. This is a generally accepted definition of Wigner matrices.

When n→∞, the empirical spectral distribution of the normalized Wigner matrix $W_{n}/\sqrt{n}$ weakly converges to the semicircular distribution, and has the following PDF:

(8) $$f^{\frac{1}{\sqrt{n}}W_{n}}\left(x\right)=\left\{\begin{array}{cc} \frac{1}{2\pi }\sqrt{4-x^{2}}, & \mathrm{if}\left|x\right|\leq 2,\\ 0, & \mathrm{otherwise}. \end{array}\right.$$

If $\eta_{k}$ denotes the *k*th moment of the semicircular law, we can obtain the following lemma. For k=0,1,2,⋯, we have [26]:

(9) $$\left\{\begin{array}{c} \eta_{2k}=\frac{2^{2k+1}}{\pi }\cdot \frac{\Gamma \left(k+\frac{1}{2}\right)\Gamma \left(\frac{3}{2}\right)}{\Gamma (k+2)}=\frac{1}{k+1}\left(\begin{array}{c} 2k\\ k \end{array}\right),\\ \eta_{2k+1}=0, \end{array}\right.$$

where Γ(·) represents Gamma function.

Suppose that $x_{1},\cdots x_{m}$ are independently identically distribution (IID) samples drawn from a n-dimensional multivariate normal population $N(\mu ,I_{n})$. Then, the sample covariance matrix is defined as:

(10) $$S_{m}=\frac{1}{m-1}\sum_{i=1}^{m}\left(x_{i}-\bar{x}\right){\left(x_{i}-\bar{x}\right)}^{T},$$

where $\bar{x}=\frac{1}{m}\sum_{i=1}^{m}x_{i}$. When *m* tends to infinity, $S_{m}\to I_{n}$ and mmnn(Sm−In)→Gn. $G_{n}$ denotes the normalized Wigner matrix.

Suppose a Wigner matrix is a symmetric matrix of N×N, and, for the sake of derivation, we set *N* = 2 m. The PDF of the eigenvalues of Wigner matrix can be calculated by [27]:

(11) $$f\left(x\right)=\frac{1}{2m}\sum_{i=0}^{2m-2}h_{i}^{2}\left(x\right)+\frac{\sqrt{2m-1}}{2\sqrt{2}m}h_{2m-1}\left(x\right)\int_{0}^{x}h_{2m-2}\left(t\right)dt,$$

where $h_{i}\left(x\right)$ is a standardized Hermitian function and $h_{i}\left(x\right)={\left(\sqrt{\pi }\cdot 2^{i}\cdot i!\right)}^{-\frac{1}{2}}\cdot e^{-\frac{1}{2}x^{2}}\cdot H_{i}\left(x\right)$, $H_{i}\left(x\right)$ is an Hermitian polynomial and $H_{i}\left(x\right)={\left(-1\right)}^{i}\cdot e^{x^{2}}\cdot \frac{d^{i}}{dx^{i}}e^{-x^{2}}$.

The spectral CDF of Wigner matrices in finite dimensions can be expressed by the equation:

(12) $$F\left(x\right)=\int_{-\infty }^{x}f\left(t\right)dt=\frac{1}{2m}\sum_{i=0}^{2m-2}\int_{-\infty }^{x}h_{i}^{2}\left(t\right)dt+\frac{1}{4m}\int_{-\infty }^{x}h_{2m-1}^{2}\left(t\right)dt.$$

For purpose of calculating the display expression in Equation (12), we need to calculate the integral term $\int_{-\infty }^{x}h_{i}^{2}\left(t\right)dt$. Bringing the expression of the standardized Hermitian function into the integral term, we can obtain:

(13) $$\int_{-\infty }^{x}h_{i}^{2}\left(t\right)dt=\int_{-\infty }^{x}\frac{1}{\sqrt{\pi }\cdot 2^{i}\cdot i!}e^{-t^{2}}H_{i}^{2}\left(t\right)dt=\frac{1}{\sqrt{\pi }\cdot 2^{i}\cdot i!}\int_{-\infty }^{x}e^{-t^{2}}H_{i}^{2}\left(t\right)dt.$$

For simplicity, $\frac{d^{i}}{dx^{i}}\Delta$ is represented by $\partial_{x}^{i}\Delta$. Thus, the Hermitian polynomial can be described as: $H_{i}\left(x\right)={\left(-1\right)}^{i}\cdot e^{x^{2}}\cdot \partial_{x}^{i}e^{-x^{2}}$. Bringing it into $\int_{-\infty }^{x}e^{-t^{2}}H_{i}^{2}\left(t\right)dt$, we get:

(14) $$\int_{-\infty }^{x}e^{-t^{2}}H_{i}^{2}\left(t\right)dt=C_{i}\left(x\right)+\sqrt{\pi }\cdot 2^{i}\cdot i!\cdot \Phi \left(\sqrt{2}x\right),\;\;\left(i\geq 2\right),$$

where $\Phi \left(x\right)$ is the CDF of the standard normal distribution and $C_{i}\left(x\right)$ is given by:

(15) $$\begin{array}{cc} C_{i}\left(x\right)= & -e^{-x^{2}}H_{i}\left(x\right)H_{i-1}\left(x\right)+{\left(-1\right)}^{i}e^{-x^{2}}\sum_{t=1}^{i-1}\left\{{\left(-1\right)}^{t}\cdot \right.\\ & \left.2^{t}\cdot \left[\prod_{s=0}^{t-1}\left(i-s\right)\right]\cdot {\left(-1\right)}^{i-t-1}\cdot H_{i-t}\left(x\right)\cdot H_{i-t-1}\left(x\right)\right\}. \end{array}$$

According to Equations (13) and (14), $\int_{-\infty }^{x}h_{i}^{2}\left(t\right)dt$ can be finally calculated as follows:

(16) $$\int_{-\infty }^{x}h_{i}^{2}\left(t\right)dt=\frac{1}{\sqrt{\pi }\cdot 2^{i}\cdot i!}\cdot C_{i}\left(x\right)+\Phi \left(\sqrt{2}x\right),\;\left(i\geq 2\right).$$

Therefore, $F\left(x\right)$ in Equation (12) can ultimately be calculated by:

(17) $$\begin{array}{cc} F\left(x\right)= & \frac{1}{2m}\int_{-\infty }^{x}h_{0}^{2}\left(t\right)dt+\frac{1}{2m}\int_{-\infty }^{x}h_{1}^{2}\left(t\right)dt+\frac{1}{2m}\sum_{i=2}^{2m-2}\left[\frac{1}{\sqrt{\pi }\cdot 2^{i}\cdot i!}\cdot C_{i}\left(x\right)+\Phi \left(\sqrt{2}x\right)\right]+\\ & \frac{1}{4m}\left[\frac{C_{2m-1}\left(x\right)}{\sqrt{\pi }\cdot 2^{2m-1}\cdot \left(2m-1\right)!}+\Phi \left(\sqrt{2}x\right)\right], \end{array}$$

where

(18) $$\int_{-\infty }^{x}h_{0}^{2}\left(t\right)dt=\int_{-\infty }^{x}\frac{e^{-t^{2}}}{\sqrt{\pi }}dt=\Phi \left(\sqrt{2}x\right),$$

and

(19) $$\begin{array}{cc} \int_{-\infty }^{x}h_{1}^{2}\left(t\right)dt & =\int_{-\infty }^{x}\frac{2t^{2}e^{-t^{2}}}{\sqrt{\pi }}dt=\frac{-1}{\sqrt{\pi }}\int_{-\infty }^{x}tde^{-t^{2}}\\ & =\frac{-1}{\sqrt{\pi }}\left(xe^{-x^{2}}-\int_{-\infty }^{x}e^{-t^{2}}dt\right)=\frac{-1}{\sqrt{\pi }}xe^{-x^{2}}+\Phi \left(\sqrt{2}x\right). \end{array}$$

## 3. Description of the Echo Detection Algorithm for Stealth Extended Targets

The electromagnetic wave emitted by the radar is reflected by the detection target to obtain the target echo signal, which not only contains useful signals characterizing the target traits, but also contains clutter and various noises. Aiming at the issue of stealth extended target detection of wideband radar signals, this paper only studies the target echo detection problem in the presence of background white Gaussian noise without considering the impact of clutter on echo due to the relatively long detection distance for most airborne warning radars that take airborne aircraft and missiles as targets.

### 3.1. Detection Method of Target Echo

For a single receiving antenna, the signal detection problem can be described by a binary hypothesis test of a null hypothesis ($H_{0}$: signal absent) and an alternative hypothesis ($H_{1}$: signal present), as follows:

(20) $$\begin{array}{c} H_{0}:\;x\left(n\right)=\omega \left(n\right)\\ H_{1}:\;x\left(n\right)=s\left(n\right)+\omega \left(n\right), \end{array}$$

where $x\left(n\right)$ is the received signal and $\omega \left(n\right)$ is the background noise. We set n=1,2,…,N, where *N* is the number of signal samples.

A common method to solve the hypotheses test in Equation (20) is the normality test [28], such as Jarque-Bera test, Lilliefors test, or Anderson-Darling test. Moreover, the signal detection performance of multi-receiving antenna is usually better than that of single receiving antenna. The binary hypothesis testing problem in Equation (20) can be transformed into a multiple hypothesis testing:

(21) $$\begin{array}{c} H_{0i}:\;x_{i}\left(n\right)=\omega_{i}\left(n\right)\\ H_{1i}:\;x_{i}\left(n\right)=s_{i}\left(n\right)+\omega_{i}\left(n\right), \end{array}$$

where i=1,2,…,M represents the number of receiving antennas.

One of the most typical approaches for solving Equation (21) is likelihood ratio test (LRT), which devises rational test statistics via likelihood functions. It is not easy to get the PDFs of $x_{i}\left(n\right)$ under $H_{0i}$ and $H_{1i}$ (denoted as $p\left(x_{i}\left(n\right)|H_{0i}\right)$ and $p\left(x_{i}\left(n\right)|H_{1i}\right)$), which results in a great difficulty in obtaining the likelihood function $p\left(x|H_{1(0)}\right)$, where x is a set of $x\left(n\right)={\left[x_{1}\left(n\right),x_{2}\left(n\right),\cdots ,x_{M}\left(n\right)\right]}^{T}$. Consequently, most of the research is based on the assumption that the received sample values $x_{i}\left(n\right)$ are Gaussian and independent. Among them, the maximum and minimum eigenvalue (MME) detection method [29] is one of the most effective signal detection algorithms. It utilizes the eigenvalues of the sample covariance matrix as the test statistic. Nevertheless, MME method discards most of the eigenvalue information, using only the maximum and minimum values of the eigenvalues, thus reducing the performance of signal detection to some extent.

**Figure 2:**
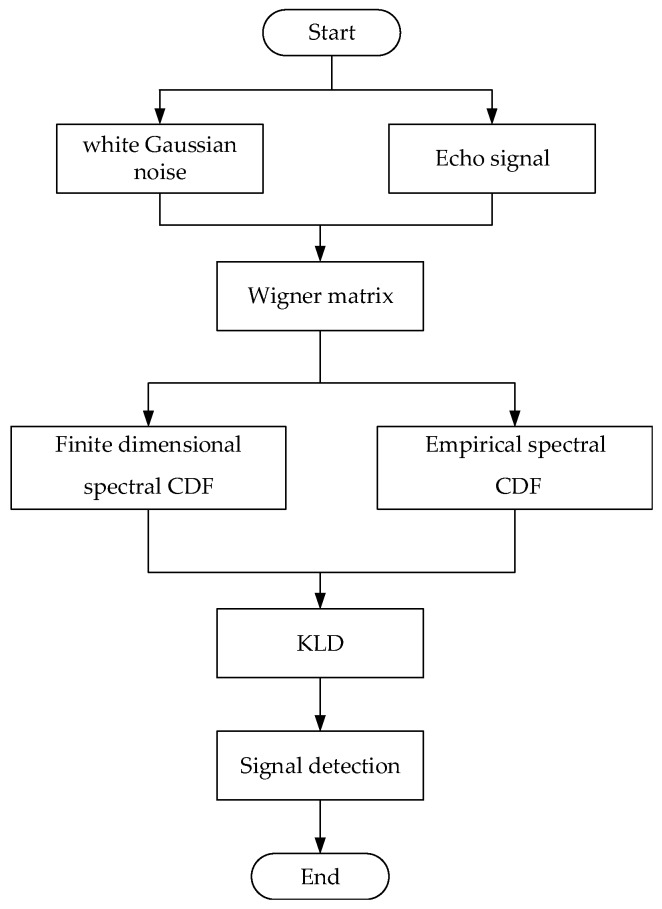
A schematic diagram of target echo detection.

Here, from the perspective of random matrix, the spectral distribution characteristic of Wigner matrices in finite dimensions is taken as the essential characteristic of white Gaussian noise, and the distance between empirical spectral CDF of observed signals and spectral CDF of Wigner matrices in finite dimensions is measured by KLD, which measures multivariate data information. The specific target echo detection process is shown in Figure 2.

### 3.2. Spectral CDF of Wigner Matrices in Finite Dimension

Assuming that the echo signal has no target, only the background white Gaussian noise, the constructed Wigner matrix is a symmetric matrix of N×N. In the light of the theoretical basis of Section 2.2, the PDF of the eigenvalues of a symmetric Wigner matrix can be formulated as:

(22) $$f\left(x\right)=\frac{1}{N}\sum_{i=0}^{N-2}h_{i}^{2}\left(x\right)+\frac{\sqrt{N-1}}{\sqrt{2}N}h_{N-1}\left(x\right)\int_{0}^{x}h_{N-2}\left(t\right)dt.$$

Please refer to Equation (11) for the meanings of the parameters in Equation (22).

Assuming that N=2n, the PDF of the symmetric Wigner matrix with N×N dimension constructed via white Gaussian noise signal can be obtained as follows [30]:

(23) $$f\left(x\right)=\frac{1}{2n}\sum_{i=0}^{2n-2}h_{i}^{2}\left(x\right)+\frac{1}{4n}h_{2n-1}^{2}\left(x\right).$$

According to Equations (12)–(17) in Section 2.2, the above PDF can be integrated to obtain the spectral CDF of Wigner matrix in finite dimension (F(x)) constructed by white Gaussian noise signal, which can be calculated as follows:

(24) $$\begin{array}{cc} F(x)= & \int_{-\infty }^{x}f\left(t\right)dt=\frac{1}{2n}\sum_{i=0}^{2n-2}h_{i}^{2}\left(t\right)dt+\frac{1}{4n}\int_{-\infty }^{x}h_{2n-1}^{2}\left(t\right)dt\\ = & \frac{1}{2n}\int_{-\infty }^{x}h_{0}^{2}\left(t\right)dt+\frac{1}{2n}\int_{-\infty }^{x}h_{1}^{2}\left(t\right)dt+\frac{1}{2n}\sum_{i=2}^{2n-2}\left[\frac{1}{\sqrt{\pi }\cdot 2^{i}\cdot i!}\cdot C_{i}\left(x\right)+\Phi \left(\sqrt{2}x\right)\right]\\ & +\frac{1}{4n}\left[\frac{C_{2n-1}\left(x\right)}{\sqrt{\pi }\cdot 2^{2n-1}\cdot \left(2n-1\right)!}+\Phi \left(\sqrt{2}x\right)\right]. \end{array}$$

For detailed explanation of the parameters, please refer to Equations (15)–(19).

### 3.3. Echo Detection of Wideband Radar Signal

The detection of stealthy extended target in wideband radar signal is described as a binary hypothesis test. When there are both target echo signal and white Gaussian noise in the echo signal, the above binary hypothesis testing problem is simply described as:

(25) $$\begin{array}{c} H_{0}:Y=Z\\ H_{1}:Y=X+Z, \end{array}$$

where $X=\left(h\times x^{T}\right)⊗I_{1\times K}$, $x\in R^{L\times 1}$ is the transmitted signal vector, $h={\left[h_{1},\ldots ,h_{p}\right]}^{T}$, $h_{i}$ is the channel coefficient from the transmitter to the *i*th receive antenna, and $I_{1\times K}={\left[1,\ldots 1\right]}_{1\times K}$. Z is an p×LK additive white Gaussian noise (AWGN) matrix. Note oversampling is used in each receive antenna with *K* samples. Let M=LK and Y can be defined as $Y=[y_{1},y_{2},\cdots ,y_{p}]$, where the sample value of echo signal $y_{i}\in R^{M\times 1}$, i=1,⋯,p is a random vector from the *M*-dimensional normal population $N_{M}\left(\mu ,S\right)$ with IID. Among them:

(26) $$\left\{\begin{array}{c} \mu =\mu \cdot I_{M\times 1},I_{M\times 1}={\left[1,1,\cdots ,1\right]}_{M\times 1}\\ S=\sigma^{2}\cdot I_{M\times M.} \end{array}\right.$$

The mean value of the sample value of the normalized echo signal and its sample covariance matrix can be obtained, which can be expressed by:

(27) $$\begin{array}{c} {\tilde{y}}_{i}=\frac{{\tilde{y}}_{i}-\hat{\mu }\cdot I_{1\times M}^{T}}{\hat{\sigma }},i=1,2,\ldots ,p\\ \mathrm{Cov}=\frac{1}{p-1}\sum_{i=1}^{p}({\tilde{y}}_{i}{\tilde{y}}_{i}^{T}). \end{array}$$

When the number of pulses is p→∞, $\mathrm{Cov}\to I_{M}$, $\sqrt{\frac{p}{M}}\left(\mathrm{Cov}-I_{M}\right)\to W_{M}$ ($W_{M}$ is a standard Wigner matrix). Let $F^{T}\left(x\right)=\sqrt{\frac{p}{M}}\left(\mathrm{Cov}-I_{M}\right)$.

Furthermore, the KLD between the empirical spectral CDF $\left(F^{T}\left(x\right)\right)$ of the random matrix constructed by the echo signal and the finite dimensional spectral CDF (F(x)) of white Gaussian noise is used to detect the extended target echo signal. The distance of the test statistic information is defined as:

(28) $$D_{KL}\left(F\|F^{T}\right)=\int_{-\infty }^{+\infty }f\left(x\right)\log\left[\frac{f(x)}{f^{T}\left(x\right)}\right]dx,$$

where *f* and $f^{T}$ denote the PDF of *F* and $F^{T}$, respectively.

According to Equations (4)–(7) in Section 2.1, Equation (28) can be further converted into:

(29) $$D_{KL}\left(U\|U^{T}\right)\approx -\sum_{p=1}^{P}\left(c_{p}-c_{p-1}\right)\log\left[\frac{U^{T}\left(c_{p}\right)-U^{T}\left(c_{p-1}\right)}{c_{p}-c_{p-1}}\right],$$

where *U* is the CDF of the uniform distribution U[0,1].

Since KLD is non-negative, the algorithm in this paper is a one-sided test problem. When the false alarm rate $P_{f}$ is constant, if the test statistic $D_{KL}\left(U\|U^{T}\right)$ is greater than or equal to the threshold value, then the null hypothesis is rejected. $P_{f}$ is the $\left(1-P_{f}\right)$-quantile of the test statistic distribution function under the null hypothesis. Since the number of samples and the distribution of the test statistic are unknown under the null hypothesis, the corresponding detection threshold can only be determined by obtaining the percentile of the empirical distribution of test statistics through the Monte Carlo method [31].

The algorithm complexity of the proposed method mainly comes from the calculation of covariance matrix Cov shown in Equation (27) and the eigenvalue decomposition of matrix Cov. The computational complexity of the sample covariance matrix is $O\left(M^{2}\cdot p\right)$. For the eigenvalue decomposition of matrix Cov, the computational complexity is $O\left(M^{3}\right)$. Consequently, algorithm complexity of this paper can be expressed by:

(30) $$O\left(M^{3}+M^{2}\cdot p\right).$$

## 4. Numerical Simulations and Performance Analysis

In this section, we provide numerical simulations to demonstrate the accuracy of the theoretical calculations as well as quantify the detection performance of the target echo detection based on KLD and Wigner matrices. This section is divided into two parts. Section 4.1 considers Figure 2 in Section 3.1 as the schematic diagram to devise the simulation and finally obtains the detection performance of the proposed method. Section 4.2 introduces the influencing factors of detection performance.

### 4.1. Comparison of Detection Performance

**Figure 3:**
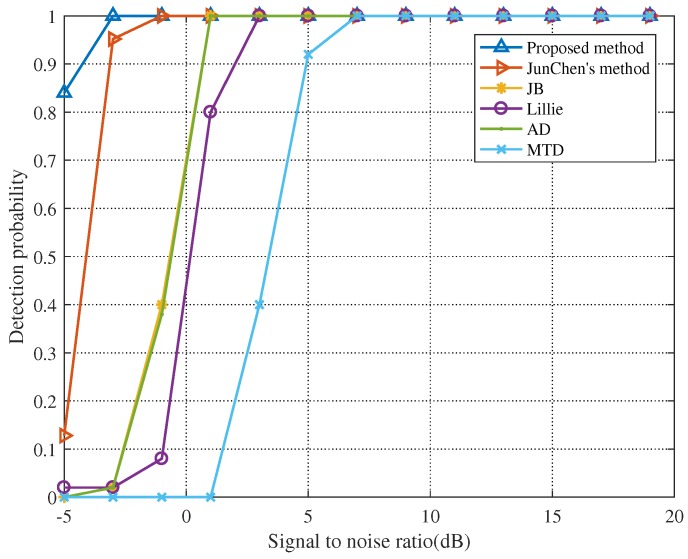
Detection probability when sampling frequency is 4.8 GHz.

Here, consider common LPI radar waveforms: Linear frequency modulation (LFM) and Poly-Phase Shift Keying (PPSK) techniques including Frank, P1, P2, P3, and P4 codes [32]. To verify the efficiency of the proposed method, Figure 3 precisely illustrates the comparison of performance between the proposed method and the three established ones, namely, Jun Chen’s method, the normality tests, and the MTD [33,34]. The normality tests are Jarque-Bera test (JB), Lilliefors test (Lillie), and Anderson-Darling test (AD). For wideband radar signals, we continuously select 10 echo pulses in the same range gate and make the width of each echo pulse as 1 μs. Each pulse may be an arbitrary LPI radar waveform, and the interval of any two pulses is stochastic and larger than the unambiguity range. The signal bandwidth is 600 MHz and the signal sampling frequency is 4.8 GHz. We set false alarm rate $P_{fa}=0.001$ and detection threshold TH=1.3226. When the test statistic $D_{KL}\left(\cdot \right)$ is greater than or equal to the detection threshold, it indicates that the signal is detected in the echo, otherwise the echo is noise.

As shown in Figure 3, when the SNR is 7 dB, the detection probability of MTD approaches 1. The detection performance of JB, Lillie, and AD are virtually the same. Even though Jun Chen’s method is superior to the above four methods, it still fails to detect signals when the SNR is −3 dB. The detection probability is close to 1 until the SNR declines to −3 dB for the proposed method. We can find that, with the increase of SNR, these methods can detect the signal, but the method proposed in this paper can obviously tackle the problem of target detection in low SNR.

**Figure 4:**
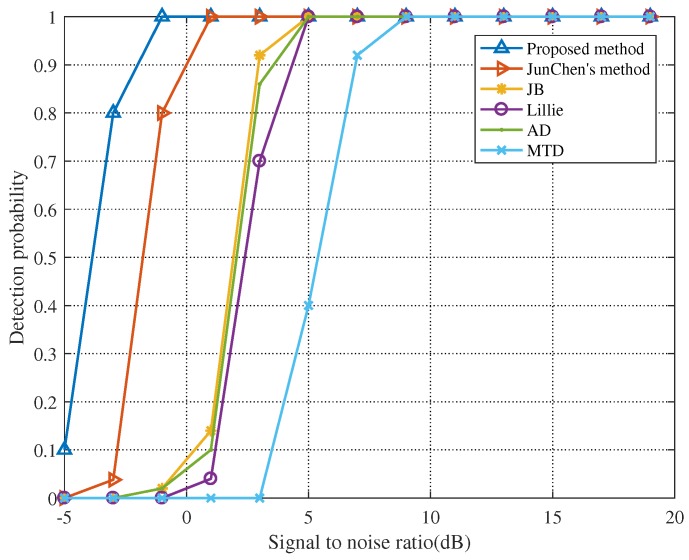
Detection probability when sampling frequency is 3 GHz.

For purpose of comparing the effect of signal sampling frequency on detection performance, we set the signal sampling frequency at 3 GHz and keep other parameters unchanged. By calculation, the detection threshold at a sampling frequency of 3 GHz is TH=1.4760. We simulated again to obtain Figure 4.

When the sampling frequency is reduced, the detection performance of all methods will decrease. For example, the detection probability is close to 1 until the SNR declines to −1 dB for the proposed method. In Figure 4, it can be seen that, even if the sampling frequency of the signal is changed, the method proposed in this paper is still superior to other detection methods.

By comparing Figure 3 and Figure 4, we can find that the sampling frequency has a certain impact on signal detection performance. For the sake of obtaining more intuitive data, we set the SNR at 3 dB and keep other parameters unchanged. The detection probabilities of the six different detection methods at different sampling frequencies are listed in Table 1 ($F_{s}$ denotes the sampling frequency of the signal).

**Figure 5:**
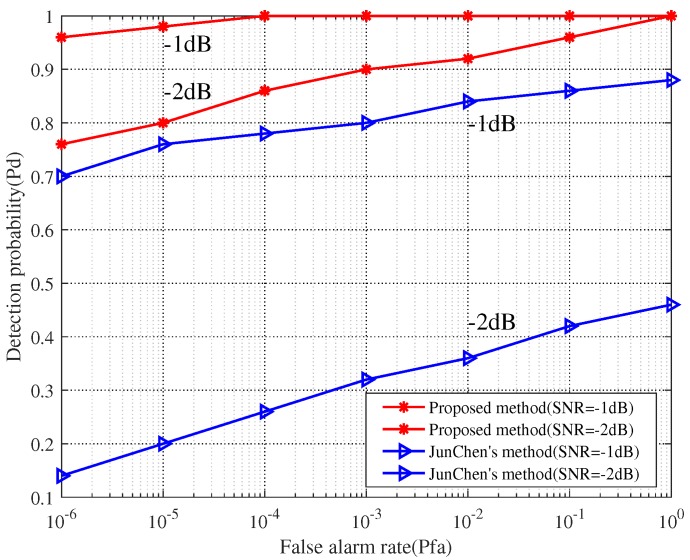
Comparison of ROC over different signal to noise ratio.

As can be seen from Table 1, the proposed method and Jun Chen’s method are relatively superior to the other methods. Figure 5 shows the receiver operating characteristic (ROC) curves of these two methods over different SNR. We set the signal sampling frequency at 3 GHz and keep other parameters unchanged.

In Figure 5, it can be seen that the false alarm rate and SNR are significantly and positively correlated with detection probability. In addition, with the same SNR and false alarm rate, the detection probability of the proposed method is higher than that of Jun Chen’s method.

**Figure 6:**
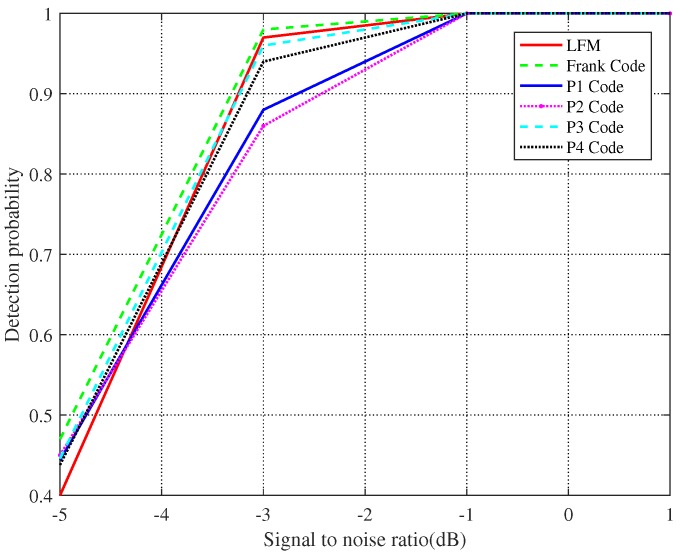
Detection performance of different LPI signals.

By comparing different detection methods, we verified the efficiency of the proposed algorithm. However, the signal we selected is random; we are not sure whether the presented algorithm is valid for ubiquitous LPI signals. To deeply investigate the robustness of the advocated method, we experimentally simulated LFM, Frank, P1, P2, P3, and P4 codes to demonstrate that the proposed algorithm has fabulous target detection performance, as shown in Figure 6.

As can be observed in Figure 6, the algorithm proposed in this paper has positive detection performance for the LPI signals listed above. Therefore, we can find that the stochastic or non-stochastic modulation of the signal has little impact on the detection of the echo signal. When the SNR is −1 dB, the detection probability of all signals approaches 1. This fully manifests whether a radar waveform has better or worse stealth performance than another radar waveform, not only associated with the waveform itself, but also to the SNR.

### 4.2. Influencing Factors of Detection Performance

Figure 3, Figure 4, Figure 5 and Figure 6 show that the SNR has a significant impact on target detection performance. Additionally, we also found that the starting sampling point (SSP) of the signal has a certain influence on target detection performance. When the echo signal is sampled, we cannot be sure that the sample must contain a useful signal, and we cannot identify the starting point of the useful signal. To gain more insight, we demonstrated our hypothesis through simulation experiments.

**Figure 7:**
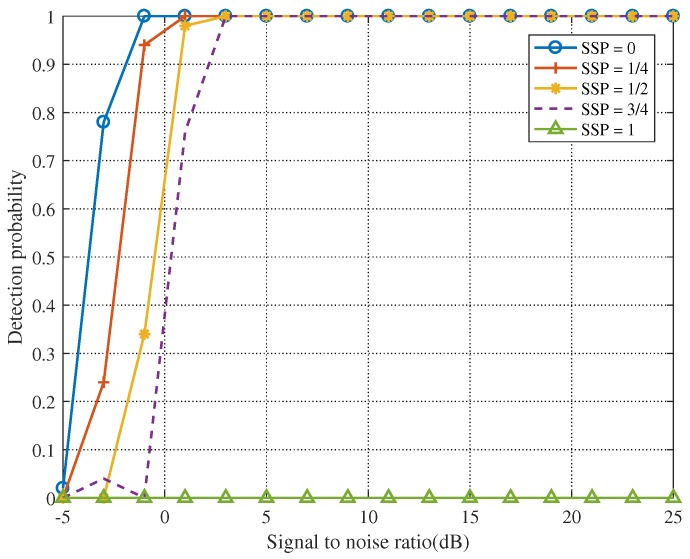
The influence of SSP on detection performance when $F_{s}$ = 3 GHz and TH=1.4760.

SSP=0 means that there is a signal in the initial sample data, SSP=1/4 means no signal in the first 1/4 of the sample data, SSP=1/2 means no signal in the first 1/2 of the sample data, SSP=3/4 means no signal in the first 3/4 of the sample data, and SSP=1 means no signal in the sample data. Figure 7 shows that the earlier is the SSP, the better is the detection performance.

**Figure 8:**
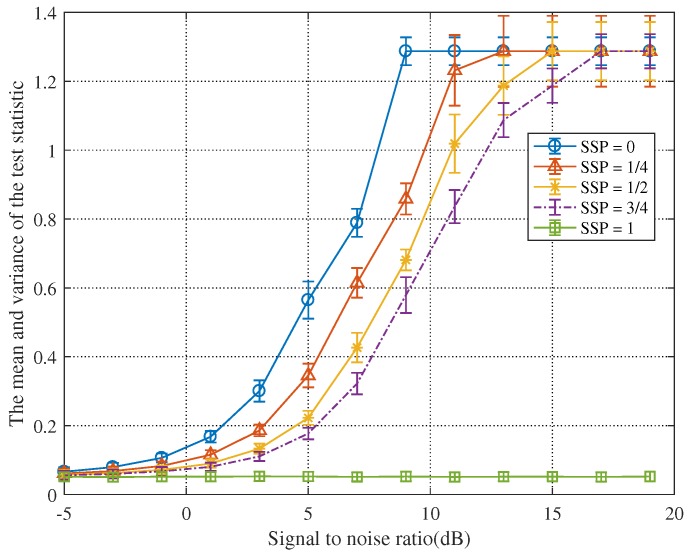
Statistical analyses of test statistics advocated by Jun Chen.

**Figure 9:**
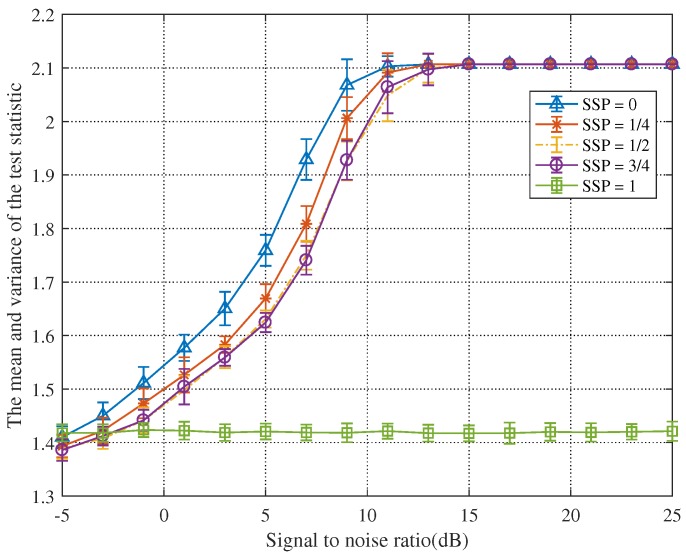
Statistical analyses of test statistics advocated in this paper.

Figure 8 and Figure 9, respectively, exemplify certain statistical analyses on the test statistics advocated by Jun Chen and the proposed method. Similarly, the parameter settings here are the same as those given in Figure 4.

Figure 8 shows that, as the SNR increases, the test statistic will continue to increase and eventually stabilize. However, when there is no signal in the first 1/2 and the first 3/4 of the data, the variance of the test statistic will not reach a constant value. These indicate that the detection method presented by Jun Chen has no steady detection performance.

Conversely, in Figure 9, when the SNR reaches 13 dB, the test statistic will tend to stabilize. As the SNR increases, the variance of the test statistic first becomes larger and then becomes smaller until it is 0. These elucidate that the detection method proposed in this paper has strong robustness. Furthermore, it can be found that the minimum value of the test statistic in the blue line is greater than the detection threshold when the SNR is −1 dB. This means that the test statistic can detect the signal when the SNR is greater than −1 dB. When only the noise is included in the first 1/4 and the first 1/2 echo signals, the test statistic can detect the echo signal when the SNR is greater than 1 dB.

## 5. Conclusions

In this paper, a new target detection algorithm is brought forward from the perspective of information theory and random matrix for stealthy extended target. This algorithm is innovatively integrated into the basic theory of Wigner matrices and KLD. Numerical results verify that the proposed algorithm has favorable detection performance and strong robustness, which enable it to secure a superiority over other alternatives. Not only does it increase the sensitivity of airborne radar, but it also provides a novel idea for designing airborne radar RFS technology.

Furthermore, the following works will be further studied in the future.

1. Containing abundant information, KLD is taken as the test statistic, whose mean and variance are studied in this paper and whose deeper content needs to be further explored in the future.
2. Figure 6 demonstrates that the proposed method has fabulous target detection performance for common LPI radar waveforms (LFM, Frank, P1, P2, P3, and P4 codes), but for advanced LPI signals, whether the proposed method can still maintain superior detection performance remains to be further studied.
3. It is widely believed that finding a balance between observation duration and detection rates is the ultimate goal of the detection of ultra-high-speed targets. Hence, it also might be of interest to devise an effective detection method of ultra-high-speed targets in a short observation time.

## Figures and Tables

**Table 1:** Signal detection performance at different sampling frequencies.

Method	$F_{s}$ (GHz)				
	0.5	1	2	3	4.8
Proposed method	0	0.64	0.9	1	1
Jun Chen’s method	0	0.24	0.88	1	1
JB	0	0	0.23	0.94	1
Lillie	0	0	0	0.66	1
AD	0	0	0.03	0.92	1
MTD	0	0	0	0	0.4
